# Closed Die Upsetting of Aluminum Matrix Composites Reinforced with Molybdenum Disulfide Nanocrystals and Multilayer Graphene, Implemented using the SPS Process—Microstructure Evolution

**DOI:** 10.3390/ma11060994

**Published:** 2018-06-12

**Authors:** Marek Kostecki, Mateusz Petrus, Jarosław Woźniak, Tomasz Cygan, Andrzej Olszyna

**Affiliations:** Faculty of Materials Science and Engineering, Warsaw University of Technology, Wołoska St. 141, 02-507 Warsaw, Poland; mateusz.petrus@pw.edu.pl (M.P.); jaroslaw.wozniak@pw.edu.pl (J.W.); tomasz.cygan@pw.edu.pl (T.C.); andrzej.olszyna@pw.edu.pl (A.O.)

**Keywords:** aluminum matrix composites, multilayer graphene, molybdenum disulfide, SPS sintering

## Abstract

New methods for producing composite materials such as SPS (Spark Plasma Sintering) are becoming more and more popular due to the ease of implementation in industrial conditions and the versatility of the materials used for processing. In order to fully exploit the potential of this method, modifications were proposed which consisted in the deliberate induction of deformation during the sintering process. The influence of the manufacturing method on the microstructure of aluminum alloy matrix composites reinforced with layered crystals in the form of nanoflakes was investigated. Composites with the addition of 10 vol % of multilayer graphene and molybdenum disulfide were prepared and their density, hardness, and the influence of the deformation ratio on the changes occurring in the microstructure were examined. The potential of the method to shape the properties of the tested composites and the strong dependence of the obtained results on the morphology of the reinforcing phase was indicated. An interesting phenomenon observed for composites with the addition of MoS_2_ during the process was the reaction of the components leading to in situ formation of the Al_12_Mo intermetallic phase.

## 1. Introduction

A permanent interest in composite materials and the development of a unique group of new materials called two-dimensional (2D) crystals have contributed to an increase in the research and application potential of composites with the addition of those prospective structures. Graphene as the monolayer of carbon atoms—a notorious 2D material—gains a number of interesting properties and a very important application of this material is found in electronics [1]. Its use as a volume component of the composite, although in theory could lead to a significant improvement of properties, in practice is economically unjustified and difficult to implement. Multilayer graphene (MLG) is becoming an increasingly popular form of graphene, which is used to improve the composite’s properties [2]. It is easier to obtain and therefore cheaper. Very thin forms of platelets containing several to dozens of layers can also be made for other layered crystals such as MoS_2_ and WS_2_ [3]. Contemporary research work suggests that the use of nanocrystals in a form similar to a monolayer, as in the case of MLG, proves to be beneficial in view of the possibility of improving the mechanical properties [4,5,6,7]. More often, in addition to structural composites, functional materials are also produced in which nanocrystals similar to 2D structures can improve their thermal [8,9,10], electrical [11], or electromagnetic [12] properties.

Composites based on aluminum alloy with the addition of solid lubricants such as graphite or molybdenum disulfide have been used for many years as light functional materials with self-lubricating properties [13]. Graphite-reinforced composites are better suited for use in atmospheric conditions because of the need for little humidity for proper delamination, while molybdenum disulfide can be used preferably in vacuum conditions [14], which makes it an interesting material for space applications. Solid lubricants are often used in composites with a much wider chemical composition (so-called hybrid composites), where the basic additions are ceramic particles that improve mechanical properties such as hardness or creep resistance, and layered crystals that provide lower frictional resistance and better workability [15].

An important issue in the design of this type of composite is the form of the layered crystals addition; more precisely, the morphology-transverse and longitudinal dimensions. Layered crystals with a decreasing transverse thickness will take the form of platelets, which will largely affect their arrangement in the volume of the composite [16]. This factor can determine the properties of the composite by providing strong anisotropy of the internal structure [17].

Another important issue for the group of composites described is the manufacturing process. In the case of multiphase materials, there is a wide spectrum of microstructure shaping possibilities. However, the low melting temperature of aluminum allows the use of classical metallurgy processes. Due to its lower costs and better process control. die compaction is currently the most popular technique for aluminum matrix composites shaping [18]. The SPS (Spark Plasma Sintering) technique, as one of the powder metallurgy methods, arouses special interest resulting from the possibility of easily implementing this method under industrial conditions. The characteristics of SPS are the fast heating rate and the effective grain size reduction [19]. Moreover, as a result of Joule-heating, we observe rapid, localized heating of the powder, breaking up the otherwise intrusive oxide layer on aluminum particles and thus speeding up the densification process [20].

Powder metallurgy, in the case of nanoparticle composites, often limits the possibility of achieving high dispersion and leaves residual porosity that is difficult to eliminate, even during process optimization [21]. A number of forming and post-processing techniques have been developed that stimulate the ability to improve the properties of metal matrix composites for specific applications. The most important are: Semisolid forging, powder forging, and hot isostatic compaction. Unfortunately, most of these processes are expensive and poorly utilized by the industry. Despite the existence of a number of solutions, fundamental research is still required, taking into account the impact of stress, strain, powders morphology, and process parameters such as temperature and time on the final product.

The authors’ experience related to obtaining composites with the participation of layered nanoparticles [4] and their influence on tribological properties [22] became an inspiration to undertake research on the possibility of improving the properties of composites with 2D particles by controlled deformation processes carried out under compacting conditions in the SPS equipment. The proposed experiment has many common features with forging/upsetting processes [23], successfully carried out for aluminum alloys of the 2xxx and 6xxx series in a conventional press. Although there are reports of an Al-Zn-Mg-Cu alloy upsetting using an SPS press [24], the idea of material upsetting for aluminum-based composites has never been realized before.

So far, this type of process has also been used to obtain the anisotropy of the magnetic properties of Nd-Fe-B magnets [25], to produce thermoelectric Ca_3_Co_4_O_9_ ceramics with a specific crystal orientation [26]. The aim of this approach was to lead to the formation of morphological or crystallographic anisotropy. For this reason the method was called Spark Plasma Texturing (SPT). Other theoretical studies on the densification kinetics of the new SPS-forging consolidation technique verified by the testing of porous ZrC specimens [27] suggested that the proposed technique can be very useful for improving the density of materials.

Considering the facts presented earlier, we decided to discuss the changes in microstructure and basic mechanical properties observed after the modified SPS consolidation processes of AA6061 alloy composites with the participation of MLG and MoS_2_ layered crystals.

## 2. Materials and Methods

**Figure 1:**
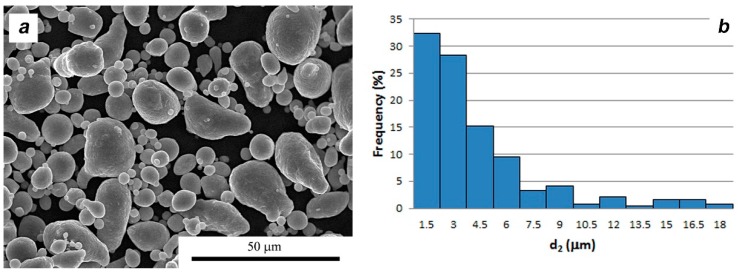
Morphology (**a**) and particle size distribution (**b**) of AA6061 powder.

Two groups of composites based on alloy AA6061 were prepared, firstly with the addition of MLG flakes and secondly with the addition of molybdenum disulfide. In order to obtain the reference material, aluminum sinters without the addition of layered crystals were also made. In each case, aluminum powder AA6061 from the Aluminum Powder Company Ltd. (Minwotrth, UK), Alpoco with the chemical composition shown in Table 1 was used as the matrix material. Larger particles of AA6061 aluminum alloy powder are mostly characterized by an elongated shape and the finer fraction of powder has a spherical shape (Figure 1a). Single large particles with an equivalent diameter exceeding 15 μm can be observed. The mean elongation (α) parameter for the statistical powder particles is 1.3.

**Figure 2:**
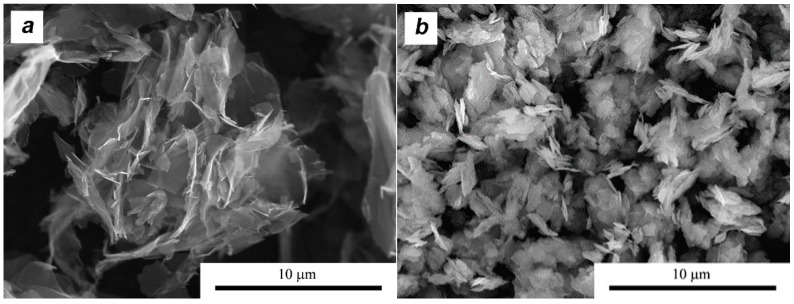
Morphology of: (**a**) multilayer graphene MLG and (**b**) MoS_2_ powders.

The MoS_2_ nanopowders from Nanostructured and Amorphous Materials, Inc. (Los Alamos, NM, USA) and the multilayer grapheme (MLG powder) trademark “Gn(12)” from Graphene Supermarket were used. In both cases the volume addition of the second phase was 10%. The morphology of the materials used is shown in Figure 1 and Figure 2.

The MLG flakes designation Gn(12) refers to the average thickness of the plates, which according to the producers is 12 nm,. Therefore, one flake comes from an average of about 40 carbon monolayers. The average lateral size of the plates is approximately 4.55 μm (1.5–10 μm). As can be seen from the data presented (Figure 2a), MLG flakes are characterized by a lateral size that is approximately 10 times larger than that of MoS_2_ flakes. One can therefore expect a significant difference in their placement already at the stage of the homogenization process. MoS_2_ crystals have a similar flake morphology (Figure 2b). They are characterized by an average lateral dimension of 480 nm and a thickness in the range of 20–40 nm.

**Figure 3:**
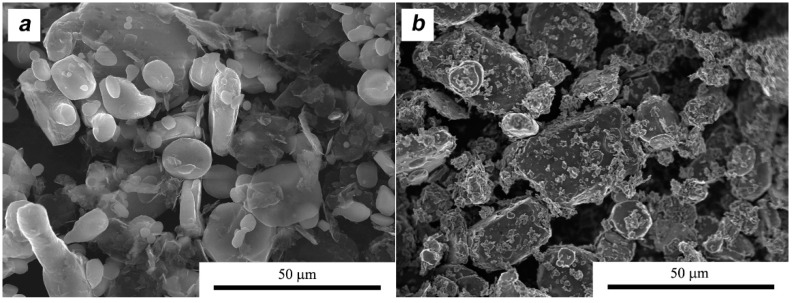
Morphology of granulated powder mixtures: (**a**) AA6061 + MLG 10 vol %; (**b**) AA6061 + MoS2 10 vol %.

After weighing, the powders were mixed in a rotary, horizontal mill in the presence of zirconium balls. The mass ratio of balls to powder was 3:1. The process was carried out with isopropyl alcohol for 8 h. The rotational speed was set at 300 rpm, enabling the smallest number of defects to be introduced into the layered crystals. Previous research [28] has shown that during mixing defoliation the formation of much thinner flakes can occur. At the same time, as shown in the Figure 3, bigger aluminum particles experience small deformations (flattening) due to collisions with ZrO_2_ balls, making a shape similar to a disk. No significant differences were found in the morphology of aluminum particles after the milling process in the presence of various additives. The mixed suspensions were dried and granulated on 0.3 mm mesh screens.

The final stage was the powder consolidation, which ran directly in the SPS device (HP D 10, FCT Systeme GmbH, Rauenstein, Germany), realizing a different strain. The process conditions are presented in Table 2. The first option included a classic, one-step process of SPS consolidation in a graphite matrix with a diameter of 20 mm, temperature T = 550 °C, and pressure of 50 MPa. This process was previously optimized to obtain material of the highest density.

The second variant was a two-stage SPT process. In the first stage, the powder was pre-sintered into a cylinder with a diameter of 20 mm under the small pressure required for the proper operation of the device and using a lower temperature. In the second stage, the previously prepared compact was subjected to the upsetting process in a larger diameter die, to allow the material to flow freely until it was completely filled. A sample with an initial diameter of 20 mm was pressed in a 30 mm die and a 40 mm die. For matrices of different diameters, a different force was used to ensure a constant pressure of 50 MPa.

**Figure 4:**
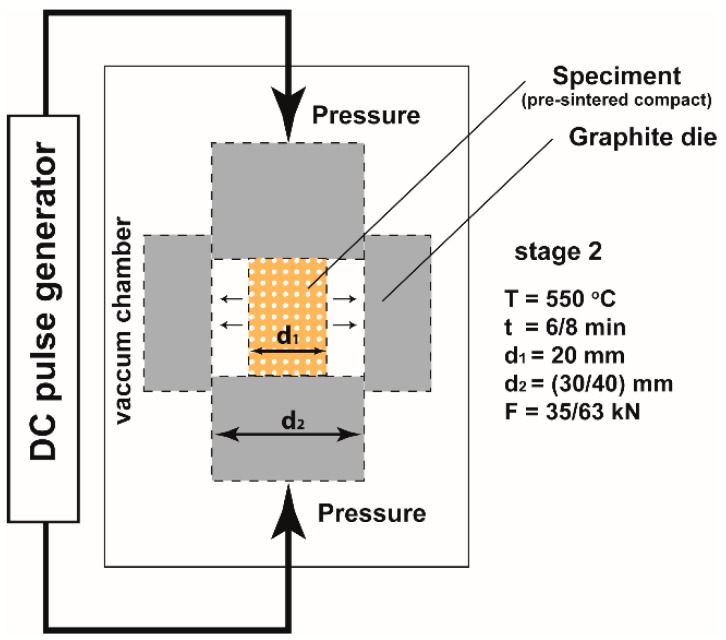
SPT processing scheme.

For simplicity, in the further part of this article we will use the deformation ratio λ = d/d_0_, meaning the ratio of the final diameter (d) of the die to the initial diameter (d_0_). For the SPS process, the deformation ratio λ = 1 and for SPT λ = 1.5 or 2. The values of the main parameters of the SPT process are listed in Table 2 and the process is schematically shown in Figure 4. For both compositions, an identical material volume was assumed for the same degree of deformation. The initial heights of the stage I samples were 22 and 30 mm according to the deformation ratio.

The obtained sinters were mechanically ground and polished first with diamond paste down to a grit size of 1 μm and finally with nanosilica suspension. In order to reveal the microstructure, samples were etched with Dix Keller reagent. The powders morphology and microstructure were observed using scanning electron microscopes (Hitachi S3500 and S5500, Japan) as well as a light microscope (NIKON Eclipse MA200, NIKON, Tokyo, Japan). Microstructures and chemical compositions of the samples were examined with a transmission electron microscope (TEM) (FEI, Hillsboro, Oregon, OR, USA) Tecnai G2 operating at 200 kV, equipped with an energy dispersive X-ray (EDX) microanalyzer and a high angle annular dark field detector (HAADF). Stereological methods were used for the precise description of powders and structures based on their planar images. The characterization of powders and grain structures was conducted for individual grains/particles and parameters such as: Equivalent average diameter (grain size): d_2_, maximal projection: d_max_, and minimal projection: d_min_. Also, the shape factor α = d_max_/d_2_ and was measured. These parameters were determined with the planimetric methods and computer image analysis (NIKON NIS-Elements; Tokyo, Japan). Fundamental properties of the obtained materials such as density (Ultrapycnometer 1000 helium pycnometer Quantachrome Instruments, Boynton Beach, FL, USA) and hardness (Vickers Hardness Tester FV-700e, Future-Tech, Kawasaki, Japan) were measured.

## 3. Results and Discussion

Upsetting in a closed die is a quite complex process from a mechanical point of view [29]. Its full understanding will involve the analysis of many process parameters such as stresses and strains present in the material along with the influence of the additive volume. This research focused mainly on microstructural issues. The addition of 10 vol % of layered crystals was chosen because of its improvement of the tribological properties, which was described in earlier works [4,22], mainly consisting of the creation of permanent tribofilm and a decrease in the wear rate. The addition of this amount also allows easy tracking of microstructural changes and comparison of test results for different types of additives with different morphologies.

### 3.1. Physical Properties

As a result of running a number of optimization processes for SPS sinters in the case of pure aluminum and the addition of a small amount of layered crystals up to 2 vol %, a high density exceeding 99% is achieved [4]. The proportion of additives exceeding 5% by volume is associated with the presence of a certain amount of pores at the interface and between the additive particles. Then the density of the composite drastically decreases. In the case analyzed, for the 10 vol % process, optimization allowed the achievement of a relative density near 95%. Density measurements for material subjected to SPT processes indicate an improvement for MLG composites up to 98.1%. However, the increase in the deformation ratio has little effect on further improvement of the density (98.2%).

**Figure 5:**
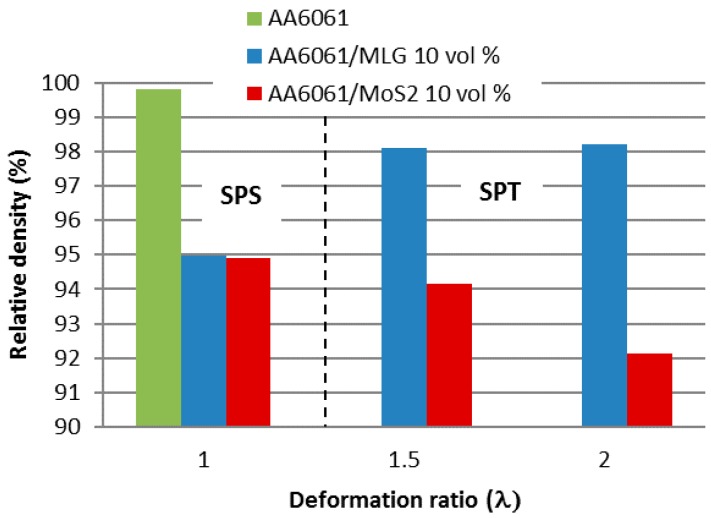
Influence of SPS and SPT processing conditions on the relative density of AA6061 matrix composites.

The addition of molybdenum disulfide completely changes the situation and the SPT process, leading to a reduction in the density of composites from 94.8% to 92.1%. Density changes resulting from the production method are shown in Figure 5.

Paradoxically, similar differences resulting from the type of additive occur in the hardness of materials and the observed behavior of the material, especially for the addition of molybdenum disulfide.

**Figure 6:**
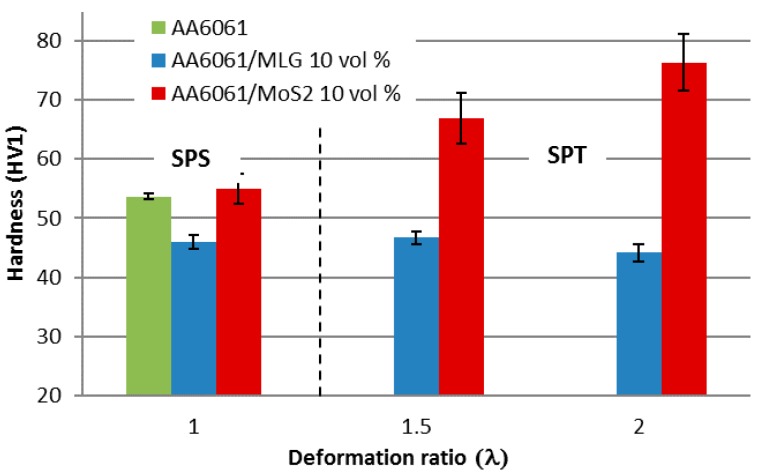
Influence of SPS and SPT processing conditions on the hardness of AA6061 matrix composites.

For the SPS process, the addition of 10 vol % of MLG flakes slightly reduces the hardness from 54 HV1 for the aluminum alloy sinter to 46 HV1 for the composite. The use of the SPT process with a low deformation ratio (λ = 1.5) has very little effect on hardness and leads to its increase to about 47 HV1. Increasing the deformation ratio slightly reduces the hardness to the level of 44.5 HV1. Composites with the addition of MoS_2_ flakes behave differently, and when using the SPT process there is an especially strong increase in the hardness of the composite. For the highest deformation ratio, the average hardness reaches 76.4 HV1. In this case there is a large standard deviation, which suggests a dispersion of the test value from the average. The examined hardness increase does not correlate with the density changes and can only be explained by microstructure analysis, which will be shown later in the article. The changes in the hardness of the tested composites are shown in Figure 6.

Because in practice such processes guarantee the inhomogeneity of stresses and strains [30] in the volume of the material being processed, one can expect far-reaching structure heterogeneities. Therefore, hardness tests were carried out on a cross-section parallel to the direction of the force acting during the sintering process. The hardness was tested from the central area to the edge of the sample on the long axis of this cross-section.

**Figure 7:**
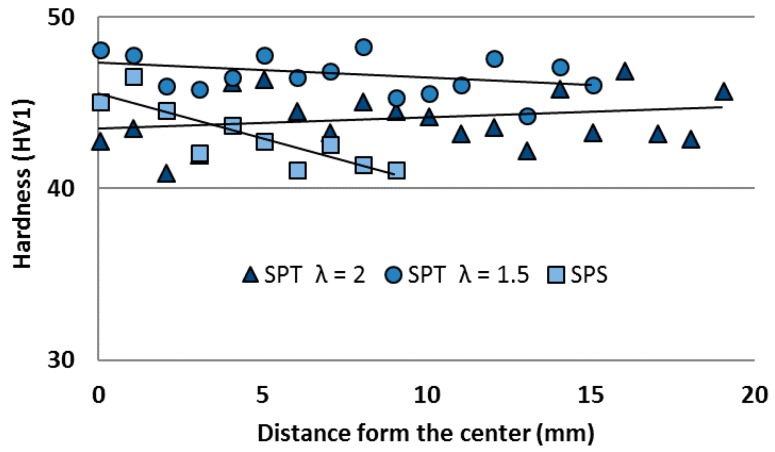
Hardness profile along part of the cross-section of AA6061/MLG 10 vol % composite.

**Figure 8:**
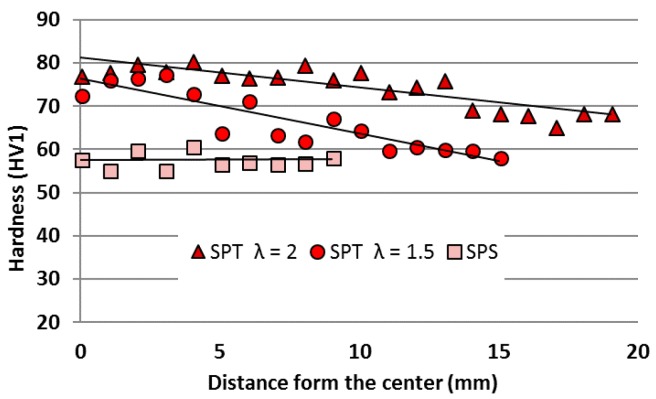
Hardness profile along part of the cross-section of AA6061/MoS_2_ 10 vol % composite.

As can be seen in the Figure 7 and Figure 8, composites with MLG content are characterized by significantly better uniformity of hardness on the cross-section than in the case of composites with MoS_2_. After the SPS process, the middle part of the sinter is slightly harder, which can be the result of the easy migration of powder particles in this area and a slight decrease in the thickness of MLG agglomerates. These changes are not visible in the microstructure. When the possibility of displacement over long distances is realized, the structure is further homogenized.

In molybdenum disulfide composite (Figure 8), a significant increase in hardness is noted, but only in the middle areas of the sample. As we move away from the center, we observe a decrease in hardness. Interestingly, for composites obtained in the SPS process, this type of heterogeneity does not occur and only reveals itself as a result of the SPT process.

### 3.2. Processing

**Figure 9:**
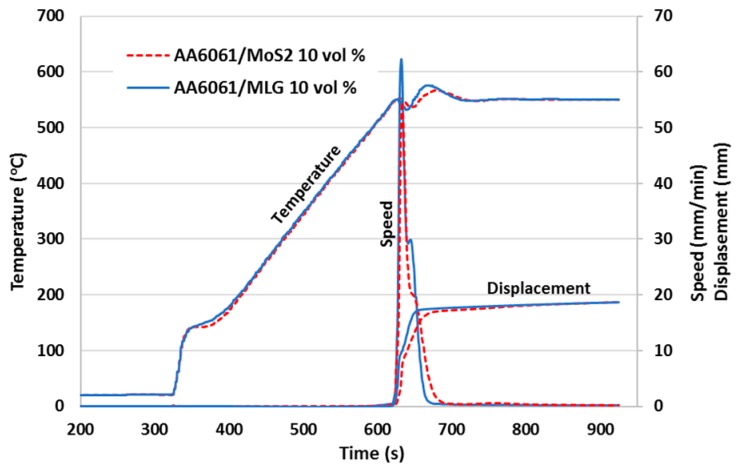
Sintering curves of AA6061/MLG 10 vol % (blue solid line) and AA6061/MoS_2_ 10 vol % (red dotted line) during the SPT (λ = 1.5) process.

**Figure 10:**
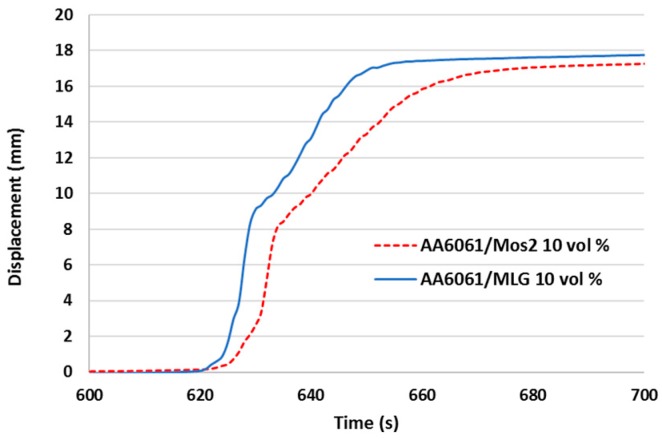
Displacement of punch for AA6061/MLG 10 vol % (blue solid line) and AA6061/MoS2 10 vol % (red dotted line) during the SPT (λ = 1.5) process.

The charts below (Figure 9 and Figure 10) show a comparison of the second stage of the SPT process for the processing rate λ = 2. In both cases, the same volume of material was used and the process was carried out with the same parameters (force and heating speed). Time differences resulting from the unequal start of the heating process after filling the chamber with protective gas were eliminated in the diagrams. Figure 9 presents a comparison of three measured values: Temperature, speed of punch, and displacement of punch for composites with various types of reinforcement (continuous and dashed curve). Due to the size of the samples (a small volume of material) differences in the curves are almost imperceptible, but enlarging the area to reflect the displacement (Figure 10) indicates that the composite with MLG is easier to deform and the punch is moved at a higher speed than the composite with MoS_2_. Similar relationships were observed for the process with λ = 1.5 and their analysis suggests that the effect is proportional to the deformation ratio.

Differences in the implementation of the SPT process can also be caused by the different ways in which current flows through the material. Depending on the electrical properties of the specimen, the current flow differs drastically and can affect temperature changes in micro-areas. In the group of additives used, we have a carbon material (multilayer graphene) which has much better electrical and thermal conductivity than the MoS2 semiconductor [31]. Although it is not possible to explicitly exclude the influence of the described factors on the formation process, the authors agree that the effective differences in the obtained properties of the composites are the result of the morphology and mechanical properties of the additives used, which will be proved during microstructure analysis. Therefore, the analysis takes into account the degree of processing and cross-sectional areas differing significantly in microstructure. A detailed analysis of microstructure images should also indicate a specific mechanism of the components’ movement (matrix and reinforcement) during SPT processes.

### 3.3. Microstructure

**Figure 11:**
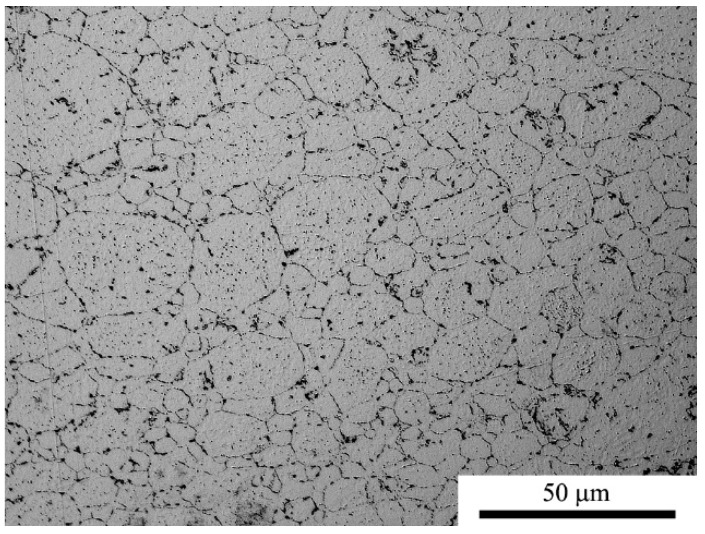
Microstructure of AA6061 (SPS sinter).

**Figure 12:**
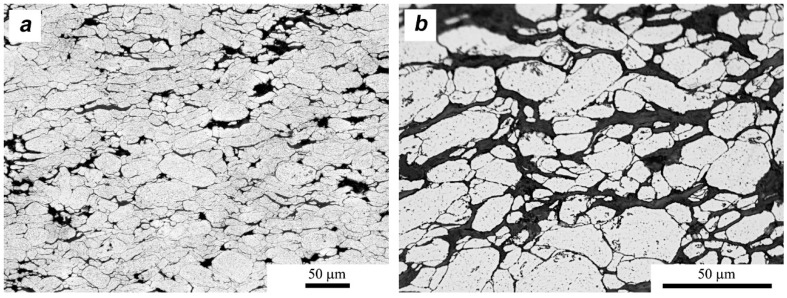
Microstructure of AA6061/MLG 10 vol % (SPS sinter). (**a**) general; **(b**) close-up view.

**Figure 13:**
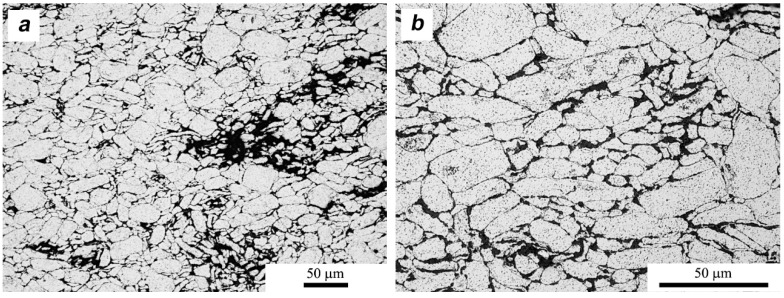
Microstructure of AA6061/MoS_2_ 10 vol % (SPS sinter). (**a**) general; (**b**) close-up view.

Figure 11, Figure 12 and Figure 13 show a series of microstructures indicating the basic differences of the composites and aluminum alloy sinters obtained in the SPS process. Due to the etching process, both intergranular aluminum boundaries and phase boundaries are visible. Bright areas are aluminum grains and dark areas represent layered crystals—MoS_2_, MLG, and the other phases present in the alloy—as well as oxides present on the surface of the powder. The aluminum sinters have an average grain size of d_2_ = 10.2 μm. On the microstructure one can observe the precipitations present in the alloy, mainly Mg_2_Si, and oxides typical for materials produced by powder metallurgy methods.

In both cases of the composite materials (Figure 12 and Figure 13), we observe a series of heterogeneities indicating the effects of the homogenization process. Flakes of layered crystals with transverse dimensions of the order of nanometers agglomerate, and the final form of agglomerates in the sinter reaches the order of micrometers. In both cases, there is also a characteristic arrangement or orientation of powder particles. MLG flakes/agglomerates with large lateral dimensions are arranged perpendicularly to the direction of the applied force, while fine MoS_2_ flakes “coat” the aluminum particles tightly and it is difficult to distinguish the preferred direction of their placement. The stereological analysis indicates that the addition of MLG did not affect the size of the aluminum grain (d_2_ = 10.3 μm), while the addition of MoS_2_ caused a reduction in the average grain size in the matrix (d_2_ = 8.8 μm) in relation to sinters without additions.

**Figure 14:**
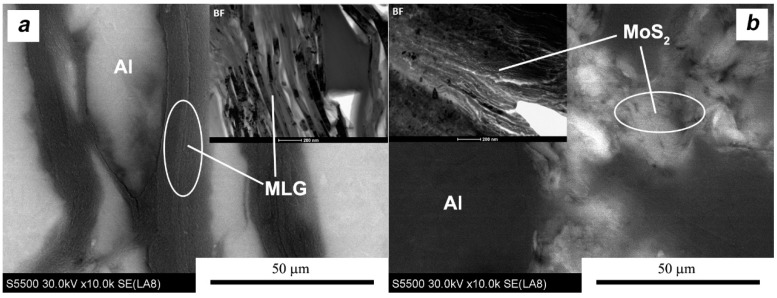
Microstructure of SPS composites, showing the arrangement of individual flakes at the borders between aluminum particles: (**a**) MLG and (**b**) MoS_2_.

The details of the platelets’ placement are important for the analysis of the SPT process, but also give an overview of the results of density measurements. Figure 14 shows distribution of individual flakes of the second phase. As can be seen, the obtained porosity is mainly the result of insufficient space filling by layered crystals which, through their morphology and the fact of the presence on the boundaries of aluminum powder particles, generate a large amount of nanopores, translating into a fairly high total porosity of 5%.

The materials obtained in the SPS process did not show any clear deviations in the microstructure over the entire cross-section. In the case of SPT sinters, a number of areas characterized by specific structural features were found as well as clear differences resulting from the location along the axis of the cross-section parallel to the direction of the pressing. For statistical purposes, in the stereological calculations, typical places from the center and near the edge areas located in cross-section were taken into account and compared.

The deformation of the pre-sintered compact induced in the SPT process causes the displacement of the composite components. Theoretical investigation also showed that the free upsetting mode generated extra shear strain components which could facilitate the mass transport within the porous specimens [27]. Because the second phase has the possibility of easy delamination, this reduces the friction of shifting crystals (particles of metal alloy powder and the layered crystals themselves). The complex state of stress in the bulk specimen means that aluminum powder particles may experience revolutions and displacements in parallel and perpendicular directions to the applied force. Due to the temperature of the process close to the melting temperature of aluminum (T_hom_ ≅ 0.8), metal powder particles can also easily change their shape during plastic deformation. In the initial phase of deformation, a large amount of layered crystals at the particle boundaries can easily change their positions and the thickness of the agglomerates changes as well.

**Figure 15:**
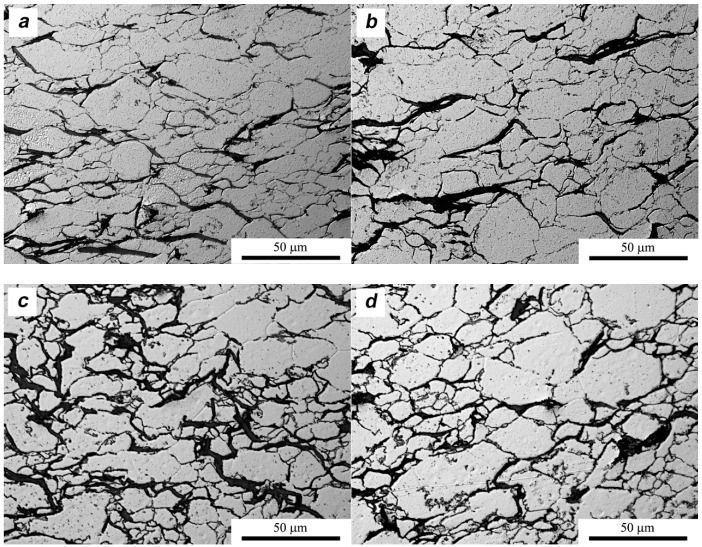
Microstructure of SPT samples AA6061/MLG 10 vol %: (**a**) center area λ = 1.5; (**b**) edge area λ = 1.5; (**c**) center area λ = 2; (**d**) edge area λ = 2.

In the composites with the addition of MLG, the displacement in the direction of tangential stresses to the direction of the force initially prevails. This is evidenced by the microstructure images from the middle areas of the sample (Figure 15a). Areas rich in MLG are characterized by a morphological texture typical of the material after rolling. The band of graphene flakes on the cross-section is arranged perpendicular to the direction of pressure. As a result of delamination, the average thickness of MLG agglomerates decreases when compared to composites produced in the SPS process from 8.6 μm to 3.5 μm. Along with the displacement, the plastic deformation of the matrix begins and the aluminum grains extend in the direction perpendicular to the pressure force, as evidenced by shape factors from the middle areas of the sample (α = 1.7). It can be assumed that reducing the thickness of the MLG agglomerates will contribute to limiting the displacements towards tangential stresses. Meanwhile, in areas distant from the specimen center (Figure 15b), the MLG agglomerates change their position in a direction parallel to the force direction. The shape coefficient of the aluminum grain (α = 1.4) indicates that the particles are more spherical. In these areas the aluminum particles can more easily rotate and the plastic deformation is lower.

With a greater degree of processing when λ = 2 in the center areas of the specimen cross-section, we observe a disturbance of the characteristic rolling texture. Although aluminum grains are still elongated (α = 1.6) (Figure 15c), graphene flakes do not show a clear orientation perpendicular to the force direction, and the average grain size of the metallic phase decreases to 7.1 μm. The reason for the reduction of aluminum grains may be a higher value of deformation and related processes of dynamic recrystallization. In areas distant from the center, the direction of the material flow also changes. The general trend is represented by the deviation of the long grain axis from the direction perpendicular to the pressing force (Figure 15d). The stereological parameters quantitatively describing the differences in the microstructure of the tested materials are presented in Table 3.

**Figure 16:**
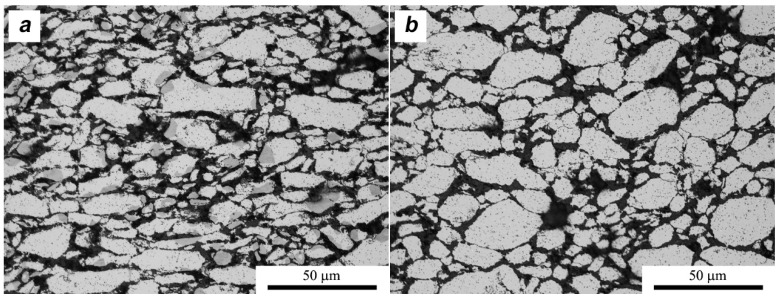

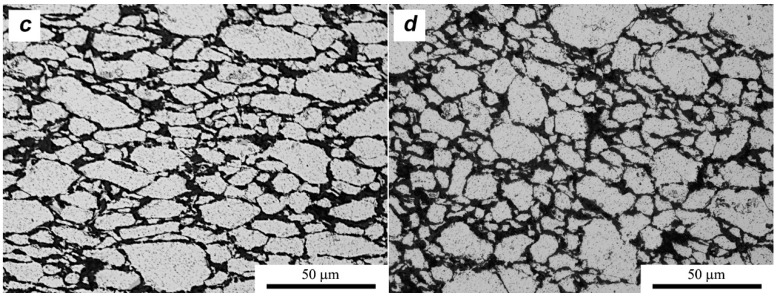
Microstructure of SPT samples AA6061/MoS_2_ 10 vol %: (**a**) center area λ = 1.5; (**b**) edge area λ = 1.5; (**c**) center area λ = 2; (**d**) edge area λ = 2.

**Figure 17:**
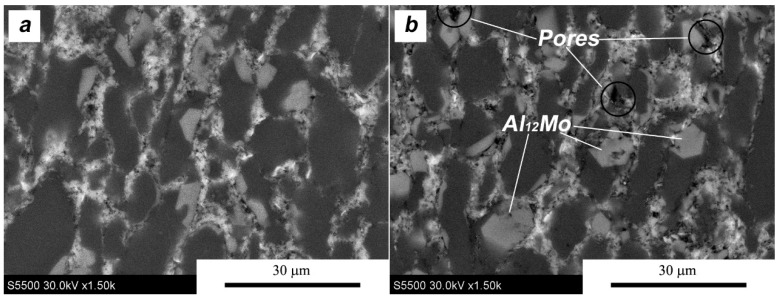
Microstructure of AA6061/MoS_2_ 10 vol % SPT samples’ center areas (BSE SEM images): (**a**) λ = 1.5; (**b**) λ = 2.

A slightly different situation is observed for composites with molybdenum disulfide (Figure 16). The deformation of the pre-sintered material causes movement in the MoS_2_ flakes relative to each other and the aluminum powder particles, but this movement is not directed. Because the flakes are not oriented in the structure, as in the case of MLG, and form an almost homogeneous barrier around Al particles, their displacement is statistically random. This entails a higher probability of rotation of the aluminum powder particles and leads to the homogenization of large areas containing the agglomerated MoS_2_ flakes visible in Figure 13a. The thickness of the MoS_2_ agglomerates observed at the grain boundary increases slightly and the boundary formed by them becomes more irregular. This is due to the fact that the platelets moving irregularly generate additional porosity, as detailed in Figure 17. The microstructure confirms the results of the density measurements presented earlier.

In the middle areas of the sinters, the grains of the matrix are flattened (Figure 16a) in the direction perpendicular to the force (α = 1.8). The value of the shape coefficient suggests that the ability to displace aluminum particles during the SPT process is more limited and the stresses present in the material in this area cause a greater deformation of the metallic matrix. On the other hand, the aluminum grains on the edge of the sinters are characterized by greater sphericity and have a smaller size (α = 1.4 d_2_ = 5.8/6.3), according to the deformation ratio (Figure 16b,d). During deformation under conditions of increased friction, smaller particles (with more regular shapes close to the sphere) of aluminum powder are easier to “push” into the edge areas, and are subjected to less deformation at the same time.

The most interesting fact related to the deformation process is the presence of bright places inside the aluminum grains in the central areas (Figure 17). Chemical analysis indicates that these areas contain elements that do not constitute alloying elements of the aluminum alloy and are characterized by fairly regular shapes in the cross-section, visible as polyhedrons that seem to “grow” from the interface inside the aluminum grain.

**Figure 18:**
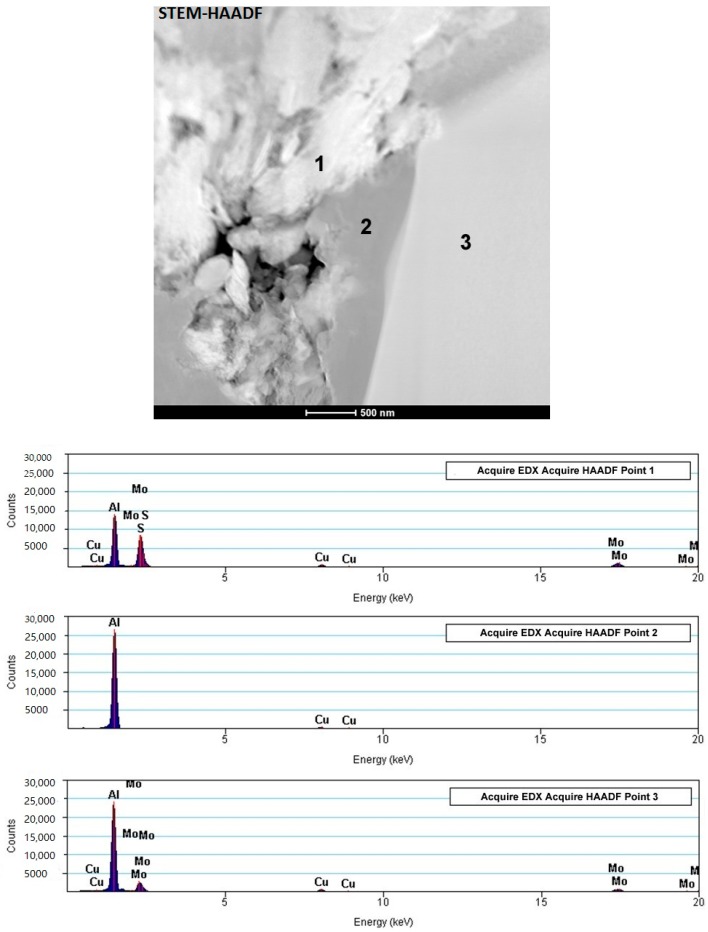
Microstructure and chemical analysis of selected points from AA6061/MoS_2_ 10 vol % SPT samples (center area λ = 1.5).

The chemical analysis of the described places is shown in Figure 18. Peaks marked as Cu come from the specimen holder. The brightest areas marked 1 represent elements such as Mo and S, which originate from the MoS_2_ phase located on the borders of the metallic powder particles. However, the presence of aluminum was also found in this place. The area marked 2 is the place inside the grain of the aluminum alloy in which the presence of aluminum was identified. In the area marked 3, also inside the aluminum alloy grain, aluminum and molybdenum were found and this is a clearly different composition compared to the remaining grain part.

**Figure 19:**
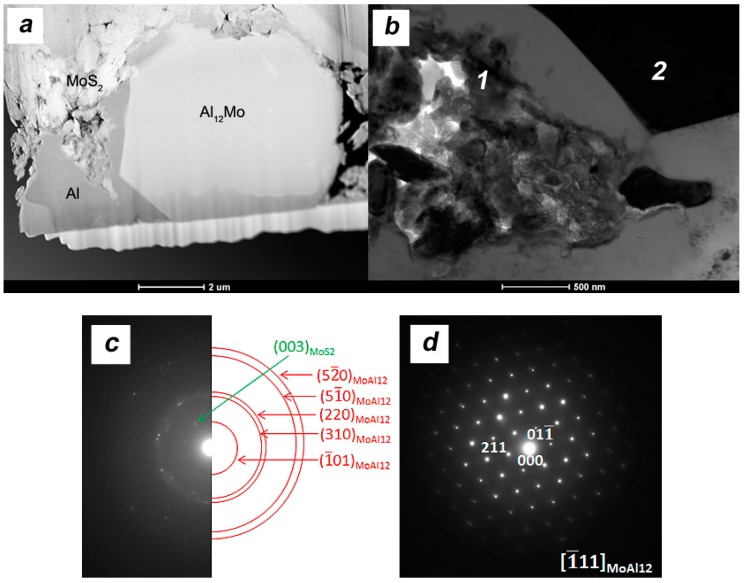
Microstructure of AA6061/MoS_2_ 10 vol % SPT samples (λ = 1.5 center area) (**a**) and (**b**) microstructure; (**c**) diffraction pattern area 1; (**d**) diffraction pattern area 2.

More precise TEM and electron diffraction studies (Figure 19) indicate the presence of a new phase in the bright areas. The Al_12_Mo phase crystallizes in composites in the form of regular areas ranging from a few to over a dozen micrometers. The crystal structure and lattice parameters of Al_12_Mo, which is the most Al-rich intermetallic phase, were determined by Adam and Rich [32] and Walford [33], as well as confirmed in other works. In equilibrium conditions, its peritectic formation temperature was determined to be 680–700 °C. The inclusions of intermetallic phases such Al_12_Mo contribute to an increase of hardness in casting Al alloys [34] and rapidly hardened aluminum alloys (RHA) [35]. Clearly, therefore, the increase in hardness observed in composites is the result of the presence of the newly formed phase. Hardness distribution and microstructure observations indicate an increased volume of Al_12_Mo phase in composites subject to stronger deformation.

At the moment, there are no studies that can confirm the possibility of this phase’s formation in composite materials in similar conditions. Although the thermal decomposition of MoS_2_ takes place at temperatures close to 1200 °C [36], it is possible that the conditions of the sintering process, in particular the high-current electric pulses and state of stress, contribute to significant deviations from the equilibrium state. We cannot exclude the presence of impurities, mainly in the form of moisture adsorbed at the powder preparation stage that affects the course of chemical reactions. There are known literature reports showing the possible reaction and degradation of MoS_2_ in the presence of water [37]. The smell of the samples immediately after sintering suggests that small amounts of hydrogen sulfide are released during the process, which are evidence of the MoS_2_ decomposition.

By analyzing the rich MoS_2_ area (Figure 19b) in the composites after the SPT process, we can see a significant fragmentation of platelets and the presence of small Al_12_Mo precipitates in the vicinity of the aluminum grains. The fragmentation of MoS_2_ is one of the factors influencing the possibility of chemical reaction with aluminum. The second factor is certainly the time of the process sustained for highly deformed composites. The longer processing time allows the diffusion necessary to create a new phase. Taking into account the influence of current pulses and pressure, temperature may rise locally and reach the melting point, which will further affect the observed phenomenon. Consideration of the presence of a new phase will certainly affect the results of relative density, but the estimated calculations indicate changes of no more than 1%.

## 4. Conclusions

Application of the SPS process to consolidate AA6061 aluminum powder or composites gives very good results in the form of high density material and good mechanical properties, which is confirmed both by literature reports and the authors’ own work. The possibility of producing useful composite materials in the powder metallurgy process is very often limited by the use of appropriate consolidation techniques and starting powders with a specific morphology. The SPS method, like other techniques, may be insufficient in the case of a high-volume fraction of the second phase particles where the main problem is to obtain a high density. During the implementation of this work, it was proposed to use a modified SPS process based on the deformation of the pre-sintered samples, similar to upsetting processes. A modified process called SPT was successfully applied.

Conducting the SPT process for composites with multilayer graphene and MoS_2_ crystals addition, representing different morphologies, led to extremely different effects. In both cases, a more or less heterogeneous strain led to different arrangements and changes in the morphology of the layered crystals platelets by defoliation and/or fragmentation.

Composites with MLG addition subjected to the SPT process exhibited an increased density and a slightly increased hardness. The microstructure of such composites was quite homogeneous, although there was a characteristic orientation of the flakes which resulted directly from their shape and axial pressure during the formation process. The process in each case led to the reduction of the MLG agglomerates thickness by their mechanical defoliation. It was found that increasing the deformation ratio does not improve the hardness, but disturbs the orientation of graphene flakes which may affect the composite physical properties such as thermal and electrical conductivity.

Composites with MoS_2_ flakes subjected to the SPT process reduced their density by the heterogeneous displacement of MoS_2_ platelets, which was primarily the result of their shape and arrangement relative to aluminum particles. On a larger scale, homogenization of the structure also occurred. However, due to the displacement of the layered crystal particles, a large porosity was generated at the nanoscale. An increase in the hardness of the sinters resulted from the in situ formation of the intermetallic phase. The intermetallic phase was formed by the reactions of the metallic matrix with the fragmented MoS_2_ crystals in the central regions of the sinter. This phenomenon was directly responsible for the increasing hardness of the composite and the variable hardness of the cross-section. Increasing the deformation ratio led to increased volume share of the created Al_12_Mo phase and hardness, but also to increased porosity.

The morphology of additives is crucial in the compaction process of the examined composites. Other factors affecting the final form of composite include the physical properties of layered crystals such as: Friction/cohesion forces within layered crystals and between components, thermal and electrical conductivity, and chemical properties leading to the reaction between components of the composite. Although they may be very important, their impact in the conducted experiment was not explained and requires further work.

## Figures and Tables

**Table 1:** Chemical composition of AA6061 powder (wt %).

Chemical Composition	Content (wt %)
Cu	0.272
Mn	0.011
Mg	**1.04**
Fe	0.111
Si	**0.634**
Zn	0.001
Cr	0.179
Ni	0.006
Ti	0.006
Al	remained

**Table 2:** Spark Plasma Sintering (SPS) and Spark Plasma Texturing (SPT) process parameters.

SPS		SPT	
		Stage I	Stage II
Temperature (°C)	550	300	550
Die diameter (mm)	20	20	30/40
Pressing time (min)	4	2	6/8
Atmosphere	Argon	Argon	Argon
Heating rate (°C/min)	100	150	100
Pressing force (kN)	16	3	35/63

**Table 3:** Stereological parameters of composite structures after SPS and SPT processes, SD: Standard deviation.

Material	SPS λ = 1		SPT λ = 1.5		SPT λ = 2	
	d_2_ (μm)/(SD)	α	d_2_ (μm) Center/Edge (SD)	α Center/edge	d_2_ (μm) Center/Edge(SD)	α Center/Edge
AA6061	10.3/(6.7)	1.4	-	-	-	-
AA6061/MLG 10 vol %	10.2/(6.7)	1.7	10.6/8.8 (7.2/6.5)	1.7/1.4	7.1/8.4 (5.1/6.3)	1.6/1.5
AA6061/MoS_2_ 10 vol %	8.8/(6.4)	1.6	8.3/5.8 (6.5/5.4)	1.8/1.5	10.1/6.3 (7.1/5.7)	1.8/1.4
